# Too Young, Too Silent: Painless Stanford Type A Aortic Dissection in a Young Adult

**DOI:** 10.7759/cureus.113473

**Published:** 2026-07-27

**Authors:** Pavithra R, Gomathi Karmegam, Satish Raja M C, Ashwini G, Manisha Shree B, Roshni Subramaniyan, Sahasyaa Adalarasan

**Affiliations:** 1 Emergency Medicine, Madras Medical College, Chennai, IND; 2 Anesthesiology and Critical Care, Madras Medical College, Chennai, IND; 3 Medical School, Madras Medical College, Chennai, IND

**Keywords:** aortic dissection, atypical, chest pain, debakey, ischemic stroke, painless, rare, stanford, young

## Abstract

Acute aortic dissection is a life-threatening vascular emergency that classically presents with severe tearing chest pain. However, painless presentations may occur, resulting in delayed diagnosis and potentially catastrophic outcomes. We report a case of a 34-year-old male who presented with sudden-onset left-sided hemiplegia, slurring of speech, facial deviation, and rapidly worsening sensorium, without any chest pain or prior comorbidities. Initial evaluation revealed threatened airway requiring intubation and mechanical ventilation. Point-of-care ultrasound and CT panaortogram demonstrated Stanford Type A aortic dissection extending from the ascending aorta to the abdominal aorta with involvement of the right common carotid artery, resulting in acute ischemic stroke and associated aspiration pneumonitis. Thrombolysis was withheld as aortic dissection is an absolute contraindication. This case highlights the importance of considering aortic dissection in young patients presenting with acute stroke and atypical presentations, as early recognition and prompt imaging are crucial to guide appropriate management and to prevent fatal complications.

## Introduction

Aortic dissection is a rare but life-threatening vascular emergency, with an estimated incidence of six in every 100,000 persons every year [1], and is much rarer in young people. It occurs when a tear develops in the intimal layer of the aorta, allowing blood to track between the intima and media and separate the layers of the aortic wall. The dissection may extend either proximally or retrogradely, impairing blood supply to vital organs. Several well-established factors have been associated with an increased risk of developing aortic dissection. Hypertension remains the most important predisposing factor, while atherosclerosis and pre-existing structural abnormalities of the aorta, including aortic aneurysm, bicuspid aortic valve, and congenital coarctation of the aorta, further contribute to disease susceptibility. Several inherited disorders affecting connective tissue integrity also confer a substantially increased risk, particularly Marfan syndrome, Ehlers-Danlos syndrome, Loeys-Dietz syndrome, and Turner syndrome, all of which are associated with varying degrees of aortic wall weakness and cardiovascular abnormalities. In addition, inflammatory vasculopathies such as giant cell arteritis have been implicated in the pathogenesis of aortic dissection. Demographic and lifestyle-related factors also influence disease occurrence, with male sex and advancing age, particularly beyond 60 years, being recognized as important non-modifiable risk factors. Transient elevations in blood pressure induced by cocaine use or strenuous resistance exercise, including heavy weightlifting, may precipitate aortic dissection in susceptible individuals. Although uncommon, pregnancy is also recognized as a potential risk factor, likely owing to the combined effects of hemodynamic stress and pregnancy-associated changes in the structural integrity of the aortic wall [2].

Aortic dissections are classified using two classification systems: the Stanford classification and the DeBakey classification [3]. 

The Stanford classification categorizes the dissections into Type A and Type B. Type A dissections involve the ascending aorta and may extend into the aortic arch and descending thoracic aorta, corresponding to DeBakey Types I and II. Type B dissections do not involve the ascending aorta and originate distal to it within the aortic arch or descending thoracic aorta, which corresponds to DeBakey Type III [4].

Acute aortic dissection is associated with a very high mortality rate (in-hospital mortality is found to be around 29.1% for Stanford Type A patients and 10.9% for Stanford Type B patients [5]),with a significant proportion of patients dying before they reach emergency medical care [6]. 

Although the typical clinical presentation is the abrupt onset of severe, “tearing” chest pain, atypical or subtle manifestations frequently result in delayed or missed diagnosis [6]. Approximately 10% of cases are painless and instead present with symptoms arising from complications caused by the dissection [7].

Stroke is one of the most serious complications associated with acute Stanford Type A aortic dissection (TAAAD). Cerebral ischemia in these patients is thought to result primarily from systemic hypotension and direct impairment of cerebral blood flow caused by the dissection [8,9]. The dissection begins with a tear in the aortic intima, allowing blood to enter the aortic wall and create a false lumen between its layers. Reduced or turbulent blood flow within this false lumen, or within the compressed true lumen, often promotes the formation of a mural thrombus. Embolization of fragments from this thrombus to the cerebral circulation can obstruct intracranial arteries, resulting in an ischemic stroke [10].

The administration of intravenous thrombolytic agents in patients with aortic dissection may lead to catastrophic complications, such as aortic rupture and progression of an intramural hematoma, resulting in stenosis of the aorta or its branches as well as distal embolization. When the dissection extends to involve the aortic root, life-threatening complications including hemopericardium and cardiac tamponade may occur, potentially leading to death [11]. Consequently, thrombolytic therapy is considered contraindicated in patients with aortic dissection. Aspiration pneumonia is an infection of the lungs caused by the entry of bacteria-laden material, such as oropharyngeal secretions, gastric contents, or particulate matter, into the lower respiratory tract. Common predisposing factors include advanced age, cerebrovascular disease (also termed post-stroke pneumonia), seizures, drug overdose, and alcohol use disorder [12]. Aspiration pneumonitis, on the other hand, is characterized by inflammation of the tracheobronchial tree and pulmonary parenchyma following aspiration [13]. Patients may present with respiratory manifestations including cough, wheeze, crackles (crepitations), dyspnoea, airway contamination, tachypnoea, tachycardia, decreased lung compliance reflected by increased airway pressures, hypoxia, and cyanosis [13].

This report describes an unusual presentation of Stanford Type A aortic dissection manifesting primarily as acute ischemic stroke in a young patient without the classical complaint of chest pain. The case highlights the diagnostic challenges and importance of early suspicion in atypical presentations of this catastrophic condition.

## Case presentation

A 34-year-old previously healthy male, an IT employee by occupation, was brought to the Emergency Department by his brother with complaints of weakness of the left upper and lower limbs for the past 1.5 hours, associated with decreased responsiveness for one hour followed by loss of consciousness.

The patient was apparently normal till the morning of presentation. Approximately an hour earlier, while travelling on a bike with his brother, he developed numbness over the left lower limb, following which he stopped the vehicle and got down. He subsequently developed weakness of the left upper and lower limbs, slurring of speech, and deviation of the angle of mouth to the right side. His sensorium progressively worsened from decreased responsiveness to unresponsiveness. There was no history of seizures, headache, vomiting, bowel or bladder incontinence, fever, chest pain, palpitations, shortness of breath, profuse sweating, bleeding manifestations, intoxication, recent trauma, or head injury.

On arrival to the Emergency Department, a primary survey was initiated. Airway assessment revealed patient had a threatened airway with poor sensorium and was intubated for airway protection and aspiration risk. He was started on mechanical ventilation. Oxygen saturation was 99% under endotracheal mechanical ventilation (ETMV) with bilateral air entry present and bilateral crepitations noted. Pulse rate was 90 beats/min, regular, normal in volume, and all peripheral pulses were equally felt. A right carotid bruit was noted on examination. Blood pressure was 142/90 mmHg in the right upper limb in supine position, with warm peripheries and capillary refill time (CRT) less than three seconds.

Neurological examination revealed GCS (Glasgow Coma Scale) E1V1M4, which later became E1VTM1 after intubation and sedation. Bilateral pupils were 3 mm, equal and reacting to light. There was paucity of movements in the left upper and lower limbs with decreased tone on the left side. Plantar response was flexor on the right side and mute on the left side. Neck stiffness was absent. Capillary blood glucose was 162 mg/dl. Head end elevation was maintained at 30 degrees. No evidence of external injury, bite marks, or spinal deformity was noted.

**Figure 1:**
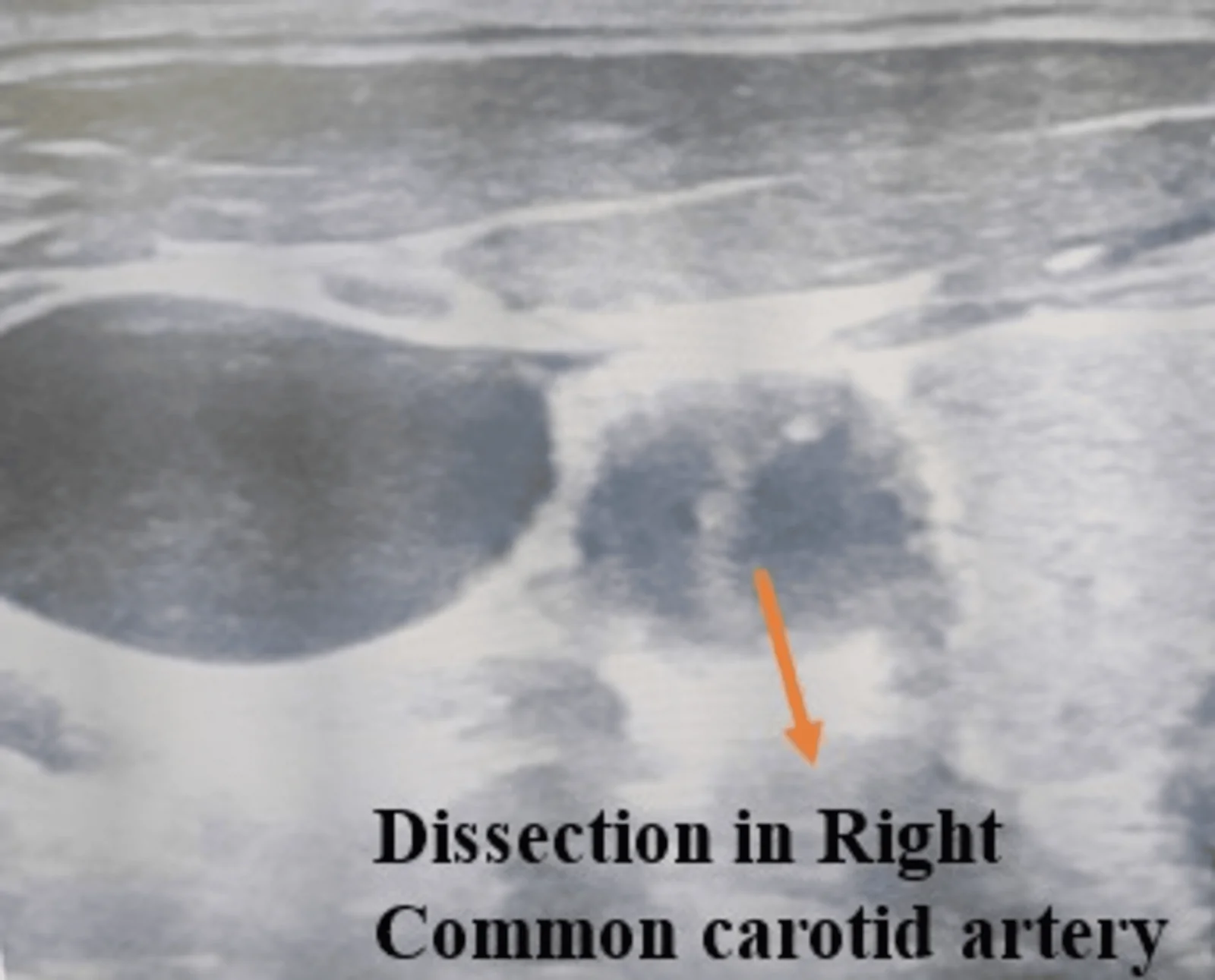
A dissection flap visualized in the right common carotid artery

Point-of-care ultrasound (POCUS) showed left ventricular systolic function (LVSF) of 60% with no regional wall motion abnormality (RWMA), pericardial effusion, clot, mitral stenosis (MS), mitral regurgitation (MR), or aortic stenosis (AS). The aortic root was dilated, measuring 4.2 cm, and the abdominal aorta measured 2.8 cm with a dissection flap noted. The dissection flap was also visualized in the right common carotid artery (Figure 1). Lung ultrasound showed a bilateral A profile except for a right posterolateral alveolar and/or pleural syndrome (PLAPS) B profile. Optic nerve sheath diameter (ONSD) was 4.5 mm. An ECG showed a normal sinus rhythm with a heart rate of 90/min, a normal axis, and no ST-T changes.

Venous blood gas (VBG) analysis revealed pH 7.39, PCO₂ 38 mmHg, HCO₃ 23 mEq/L, and lactate <2 mmol/L. Foley’s catheterization drained 250 mL of clear urine.

Secondary survey revealed no history of allergy, prior surgeries, medication use, previous similar episodes, transient ischemic attack (TIA), syncope, IV drug abuse, or illicit drug consumption. The patient had no known comorbidities

On general examination, the patient was afebrile, moderately built, and under sedation and paralysis on ETMV. Blood pressure measurements in all four limbs were right upper limb, 140/90 mmHg; left upper limb, 134/82 mmHg; right lower limb, 124/96 mmHg; and left lower limb, 142/96 mmHg. Pulse rate was 90/min, regular, normal in volume, and equally felt in all four limbs. Respiratory rate was 16/min and SpO₂ was 98% under ETMV.

Detailed CNS examination showed normal muscle bulk bilaterally with decreased tone in the left upper and lower limbs. Paucity of movements was noted on the left side and power could not be assessed. Angle of mouth deviation to the right side was present. Cardiovascular examination revealed normal S1 and S2 with right carotid bruit and no murmurs. Respiratory system examination showed bilateral air entry without added sounds.

Laboratory investigations revealed normal hemoglobin levels, mildly elevated total leukocyte count, normal platelet count, blood urea, serum creatinine, sodium, and potassium levels. CT imaging of the brain showed no evidence of intracranial hemorrhage, mass effect, or midline shift, with preserved gray-white matter differentiation and normal ventricular and cisternal anatomy. CT panaortography revealed a Stanford Type A aortic dissection with an intimal flap extending through the ascending aorta, aortic arch, descending thoracic aorta, and abdominal aorta, with further extension into the right common carotid artery (Table 1).

**Table 1:** Routine Laboratory Investigations and Vital Signs g/dL: grams per deciliter; cells/mm³: cells per cubic millimeter; mg/dL: milligrams per deciliter; mEq/L: milliequivalents per liter; mmol/L: millimoles per liter; mmHg: millimeters of mercury; beats/min: beats per minute; breaths/min: breaths per minute.

Investigation / Vital Sign	Patient Value	Reference Value
Hemoglobin	12.9 g/dL	13.0-17.0 g/dL (adult male)
Total White Blood Cell Count	11,600 cells/mm³	4,000-11,000 cells/mm³
Platelet Count	2,29,000/mm³	1,50,000-4,50,000/mm³
Blood Urea	26 mg/dL	15-40 mg/dL
Serum Creatinine	0.9 mg/dL	0.7-1.3 mg/dL
Serum Sodium	136 mEq/L	135-145 mEq/L
Serum Potassium	4.2 mEq/L	3.5-5.0 mEq/L
Capillary Blood Glucose	162 mg/dL	70-140 mg/dL*
Venous Blood Gas pH	7.39	7.31-7.41
Partial Pressure of Carbon Dioxide (PCO₂)	38 mmHg	41-51 mmHg
Bicarbonate (HCO₃⁻)	23 mEq/L	22-29 mEq/L
Lactate	<2 mmol/L	0.5-2.2 mmol/L
Pulse Rate	90 beats/min	60-100 beats/min
Blood Pressure (Right Upper Limb)	142/90 mmHg	<120/80 mmHg
Blood Pressure (Left Upper Limb)	134/82 mmHg	<120/80 mmHg
Blood Pressure (Right Lower Limb)	124/96 mmHg	Comparable to upper limb; systolic may be 10-20 mmHg higher
Blood Pressure (Left Lower Limb)	142/96 mmHg	Comparable to upper limb; systolic may be 10-20 mmHg higher
Respiratory Rate	16 breaths/min	12-20 breaths/min
Peripheral Oxygen Saturation (on Endotracheal Mechanical Ventilation)	98%	95-100%

Based on the patient's clinical features and radiological findings, a probable diagnosis of acute cerebrovascular accident involving the right middle cerebral artery territory was made, presenting with left-sided hemiplegia and left upper motor neuron facial palsy. The stroke was considered to be secondary to thrombotic occlusion resulting from a Stanford Type A aortic dissection, with concurrent aspiration pneumonitis.

Following evaluation, thrombolysis was withheld as aortic dissection was identified as an absolute contraindication. Patient was continued with intubation and mechanical ventilation in view of a poor GCS score. He was administered ceftriaxone 1 g intravenously twice daily as advised treatment, pantoprazole 40 mg intravenously stat, and ondansetron 4 mg intravenously stat. Continuous blood pressure monitoring was done while awaiting definitive surgical intervention. Despite the intensive supportive management, the patient's clinical condition progressively deteriorated, and he was declared dead on the fourth day of hospitalization before surgical repair could be performed.

## Discussion

Aortic dissection is a rare disease, with an estimated incidence of six in every 100,000 persons every year [1], and is much rarer in young people. Several factors associated with an increased risk for developing the disease include hypertension, pre-existing structural abnormalities of the aorta, inherited disorders affecting connective tissue integrity such as Marfan syndrome, Ehlers-Danlos syndrome, and Loeys-Dietz syndrome, and demographic factors such as male sex and increasing age, particularly beyond 60 years [2].

Increasing age is an important predisposing factor for acute aortic dissection, with most cases occurring between 40 and 70 years of age, particularly among those aged 50 to 65 years, who account for nearly 75% of reported cases. However, the present case involved a 34-year-old male, highlighting the need to consider rare but potentially fatal etiologies such as aortic dissection when evaluating stroke in young adults [6].

Aortic dissections are commonly classified using two major classification systems: the Stanford classification and the DeBakey classification [3]. Both systems are based primarily on the involvement of the ascending aorta.

The Stanford classification, which is the system most commonly applied in clinical practice, categorizes aortic dissections into Type A and Type B. Type A dissections involve the ascending aorta and may extend into the aortic arch and descending thoracic aorta, encompassing DeBakey Types I and II. The initial intimal tear can occur at any location within this portion of the aorta. Conversely, Type B dissections do not involve the ascending aorta and originate distal to it within the aortic arch or descending thoracic aorta, corresponding to DeBakey Type III [4].

The DeBakey classification offers a more detailed anatomical description based on the extent of the dissection [14]. Type I dissections arise in the ascending aorta and propagate at least as far as the aortic arch. They are more frequently seen in patients younger than 65 years and carry the highest mortality risk, with an estimated increase in mortality of approximately 1% per hour during the acute phase. Type II dissections are confined to the ascending aorta and are typically encountered in older patients with hypertension and underlying atherosclerotic disease. Type III dissections originate distal to the left subclavian artery within the descending thoracic aorta and are further divided into Type IIIa, which extends to the level of the diaphragm, and Type IIIb, which continues beyond the diaphragm into the abdominal aorta [4].

The occurrence of aortic dissection is closely linked to several well-established risk factors, including hypertension, atherosclerotic disease, male sex, connective tissue disorders such as Marfan syndrome and Ehlers-Danlos syndrome, and congenital abnormalities such as a bicuspid aortic valve. Acute aortic syndromes most commonly present with the abrupt onset of severe, “tearing” chest pain that typically radiates to the back. Nevertheless, despite this classic presentation, establishing the diagnosis may be challenging because some patients exhibit atypical or subtle clinical manifestations, as demonstrated in the present case [4].

Management of aortic dissection is primarily determined by the Stanford classification and the extent of aortic involvement. In Stanford Type A dissections, definitive treatment generally consists of surgical excision of the affected segment of the ascending aorta, with or without replacement of the aortic arch, followed by reconstruction using a synthetic vascular graft. When the dissection affects the structural integrity of the aortic valve apparatus, valve repair may also be required. If the dissection extends into the major branches of the aortic arch, including the brachiocephalic artery, left common carotid artery, or left subclavian artery, these vessels may require reimplantation into the graft. In patients with extensive Type A dissections involving the descending thoracic or abdominal aorta, staged surgical intervention may be necessary [3].

In comparison, uncomplicated Stanford Type B dissections are usually treated with conservative medical management, which has been associated with favorable survival outcomes when optimally implemented. Initial therapy aims to achieve strict control of blood pressure and heart rate, most commonly with intravenous beta blockers such as labetalol, while calcium channel blockers serve as alternative agents when indicated. This approach is intended to reduce aortic wall shear stress and limit further propagation of the dissection. Such treatment is ideally initiated in an intensive care unit, where patients can undergo continuous hemodynamic monitoring [3].

Radiological imaging is essential for evaluating the morphology, classification, and extent of an aortic dissection, thereby facilitating appropriate management planning. Pleural effusions are a common associated finding and often increase in volume during the acute stage of the disease [3,15].

Chest radiography may occasionally appear normal; however, several findings can suggest aortic dissection. These include widening of the mediastinum, double or irregular aortic contour, and inward displacement of atherosclerotic calcification away from the aortic margin. Depending on the underlying etiology, signs of mediastinal or periaortic hematoma may also be present, including obscuration of the aortic knob, opacification of the aortopulmonary window, deviation of mediastinal structures such as the trachea or esophagus, increased thickness of the paratracheal stripe, and left-sided apical capping [3,16-19].

CT, particularly computed tomography angiography (CTA) with arterial contrast enhancement, is considered the imaging modality of choice for diagnosing and classifying aortic dissection, while also evaluating distal complications. CTA has reported sensitivity and specificity approaching 100% [20,21]. Because thoracic dissections may extend into the abdominal aorta and iliac vessels, CT imaging of the abdomen and pelvis is often performed simultaneously to evaluate for mesenteric or visceral malperfusion.

Contrast-enhanced CTA provides excellent anatomical detail and may demonstrate features such as an intimal flap, formation of true and false lumens, aortic dilatation secondary to aortic insufficiency, intramural hematoma, Mercedes-Benz sign in triple-barrel dissections, windsock sign, and complications associated with dissection [3,20-24].

In chronic dissections, the intimal flap tends to appear thicker and straighter compared to that seen in acute dissections [3].

Surgical or endovascular treatment is generally indicated only in patients who develop complications, including aortic rupture, renal failure, visceral or limb ischemia, persistent pain despite medical therapy, or hypertension that remains uncontrolled with medication [3].

The Bentall procedure is a surgical technique performed for patients with aneurysms involving the ascending aorta or aortic root associated with aortic valve pathology. The operation involves replacement of both the proximal ascending aorta and the aortic valve using a composite valved graft [25,26]. The implanted valve may either be mechanical or bioprosthetic in nature.

This procedure is considered the surgery of choice in acute Type A aortic dissections when there is aortic root dilatation greater than 4.5 cm, presence of an intimal tear, or underlying connective tissue disorders. However, despite its effectiveness, the Bentall procedure has certain drawbacks, including the need for lifelong anticoagulation and an increased risk of complications such as stroke, bleeding, infective endocarditis, and hemolysis [25,27].

Although antithrombotic therapy is absolutely contraindicated in patients with aortic dissection [28], some studies have reported that selected patients with acute ischemic stroke secondary to aortic dissection who received thrombolytic therapy were subsequently managed successfully without major complications [10].

While our case presented with stroke, one of the common neurological complications of aortic dissection, Lauren et al. [29] described a patient whose dissection was not obvious at first because the early findings were nonspecific, with bradycardia, weakness, and a pulsatile neck swelling rather than classic chest pain or neurological symptoms. The key highlight of this case is how aortic dissection can present with subtle, misleading physical signs.

Ramy Mando et al. [30] described an extreme masquerade case in a patient whose dissection was not obvious at first because the early findings were nonspecific, with bradycardia, weakness, and a pulsatile neck swelling rather than classic chest pain. They emphasized how aortic dissection can present with subtle, misleading physical signs.

Utkarsh Khandelwal et al. [31] presented a case where the aortic dissection manifested with an unusual, visible, palpable supraclavicular pulsatile swelling, along with dull chest pain. This indicates that even a severe disease such as aortic dissection can be discovered from a rare external physical sign on examination.

Another case report by Alichetty et al. [32] talked about Type B aortic dissection with complicated multiorgan involvement, which was notable for abdominal symptoms, renal failure, bowel involvement, and later neurologic complications; its key takeaway is the multi-organ nature of the disease and the severity that can emerge when diagnosis is delayed.

Recent advances in the management of aortic dissection have focused on improving perioperative outcomes while reducing malperfusion and surgery-related mortality. One notable innovation is the “branch-first” technique, which was developed to avoid prolonged periods of circulatory arrest during total aortic arch replacement. In this approach, the head vessels are sequentially clamped and reconstructed using a specially designed trifurcated graft, thereby maintaining continuous cerebral perfusion throughout the procedure [33,34]. Another important advancement is the increasing use of the Ascyrus Medical Dissection Stent (AMDS; Artivion, Inc., Atlanta, GA, USA) in selected centers. This hybrid device consists of a partially covered proximal Teflon graft combined with a distal cylindrical nitinol frame positioned within the aortic arch [33,35]. The nitinol component helps stabilize the intimal dissection flap, promotes thrombosis of the false lumen, and facilitates remodeling of the aortic wall, thereby potentially reducing distal anastomotic re-entry tears and the future need for aortic reintervention.

## Conclusions

Aortic dissection is a life-threatening vascular emergency that can occasionally present with atypical manifestations such as acute ischemic stroke without the characteristic chest pain, making diagnosis challenging. This case highlights the importance of maintaining a high index of suspicion for aortic dissection in young patients presenting with sudden neurological deficits, especially when associated with unusual examination findings or rapidly worsening sensorium.

Early bedside assessment, prompt imaging, and multidisciplinary management are crucial in preventing fatal complications and avoiding inappropriate therapies such as thrombolysis, which may significantly worsen outcomes in patients with aortic dissection. Timely recognition of this rare presentation can aid in reducing morbidity and mortality associated with this catastrophic condition.
